# Small-caliber cap-assisted identification of an exposed vessel and direct clipping for colonic diverticular bleeding

**DOI:** 10.1055/a-2921-6422

**Published:** 2026-08-27

**Authors:** Nobutaka Doba, Masanobu Someya, Koki Goto, Ryo Yonamine, Shinzo Abe, Shin Maeda

**Affiliations:** 1 Department of Gastroenterology 36998 Yokosuka City HospitalYokosukaKanagawaJapan; 2 Gastroenterology DivisionYokohama City University, School of MedicineYokohamaKanagawaJapan

**Fig. 1:**
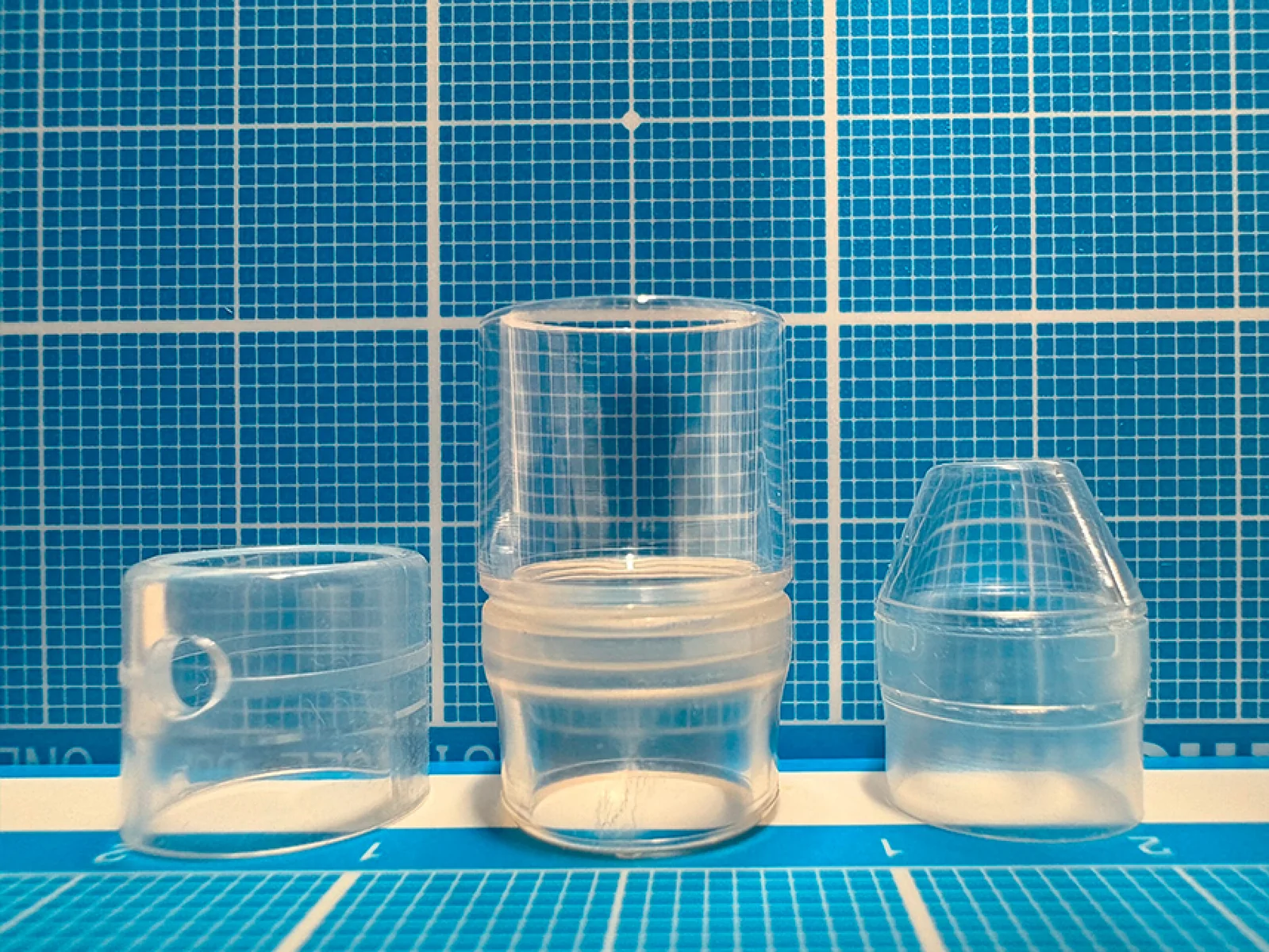
Comparison of transparent caps. Left: a standard transparent cap (distal extension length, 4 mm; tip opening diameter, 13.4 mm). Center: a long transparent cap (distal extension length, 12 mm; tip opening diameter, 13.5mm) used during the initial examination and treatment. Right: a small-caliber transparent cap (distal extension length, 5mm; tip opening diameter, 4 mm) used for the identification of the exposed vessel and direct clipping.

Colonic diverticular bleeding is one of the major causes of acute lower gastrointestinal bleeding. 1​ Although direct clipping is considered an effective endoscopic hemostatic technique, 2​ identification of an exposed vessel within a diverticulum is often challenging because of the narrow diverticular orifice and unstable endoscopic visualization. Several techniques using a long hood have been reported to facilitate the identification of the bleeding point and direct clipping 3​ ​4​ ( Fig. 1 ).

A 78-year-old woman was admitted to our hospital with hematochezia. Colonoscopy after bowel preparation revealed non-bloody intestinal fluid throughout the colon; however, an adherent coagulum was identified within a single diverticulum in the ascending colon, suggesting the bleeding source. Irrigation did not induce active bleeding. Careful observation using a long transparent cap and retroflexion failed to identify an exposed vessel within the diverticulum. Therefore, indirect clip closure of the diverticular orifice was performed ( Video 1 ).

**Fig. 2:**
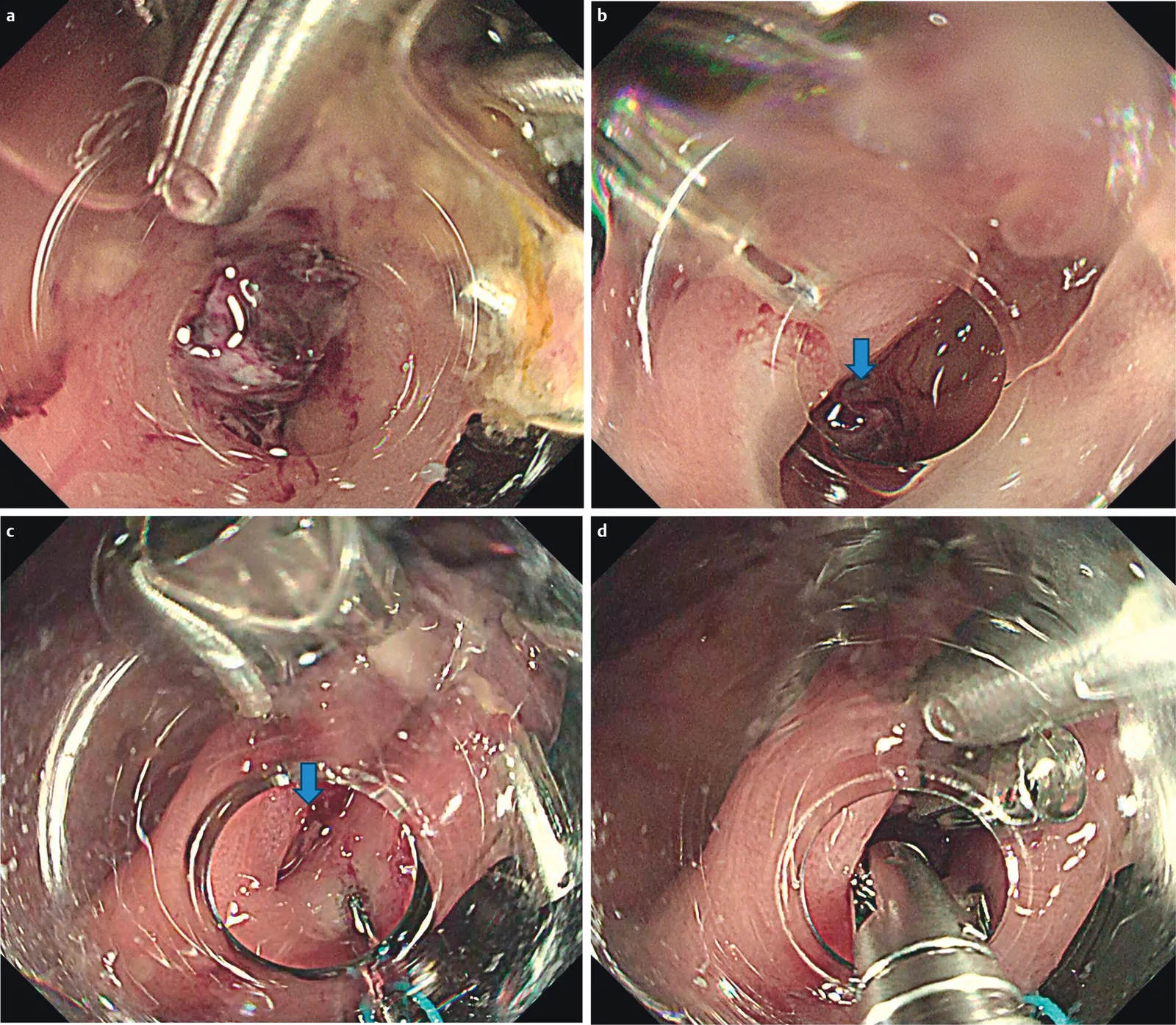
Endoscopic findings and treatment. ( **a** ) The suspected bleeding diverticulum after three sessions of indirect clip closure. An adherent coagulum remained within the diverticulum despite adequate bowel preparation. ( **b** ) After removal of the coagulum by suction through a small-caliber transparent cap, insertion of the cap tip into the diverticulum enabled the visualization of a pulsating exposed vessel. ( **c** ) A clip combined with a loop-assisted traction device was used to maintain opening of the diverticular orifice, facilitating the stable visualization of the exposed vessel. ( **d** ) Two clips were directly applied to the exposed vessel, successfully achieving hemostasis by direct clipping. The arrow indicates the exposed vessel.

**Video 1** Small-caliber cap-assisted visualization and direct clipping for colonic diverticular bleeding. Video URI:


However, hematochezia subsequently recurred, and two additional sessions of indirect clipping failed to achieve hemostasis. Repeat colonoscopy was therefore performed with the intention of direct clipping. To facilitate intradiverticular observation, a transparent cap with a 4-mm distal opening was attached to the tip of the endoscope ( Fig. 1 ). During repeat examination, coagulum was again identified only within the previously treated diverticulum ( Fig. 2a ). After removal of the coagulum by suction through the cap, insertion of the cap tip into the diverticulum enabled the visualization of a pulsating exposed vessel ( Fig. 2b Video 1 ).

To maintain stable visualization, the edge of the diverticulum was grasped using a clip combined with a loop-assisted traction device and pulled toward the opposite colonic wall, as previously reported. 5​ This maneuver maintained continuous opening of the diverticular orifice and facilitated the stable insertion of the cap tip into the diverticulum, thereby enabling the clear visualization of the exposed vessel ( Fig. 2c ). Two clips were then directly applied to the exposed vessel, successfully achieving hemostasis by direct clipping ( Fig. 2d Video 1 ). No recurrent hematochezia occurred thereafter, and the patient was discharged uneventfully.

This case highlights the potential utility of a small-caliber transparent cap for stable intradiverticular observation, the identification of the exposed vessel, and direct clipping in difficult cases of colonic diverticular bleeding. In addition, traction-assisted opening of the diverticular orifice was useful for maintaining visualization during the procedure.

Endoscopy_UCTN_Code_CCL_1AD_2AF

Endoscopy_UCTN_Code_TTT_1AQ_2AZ
